# Oxidative Stress in Assisted Reproductive Techniques, with a Focus on an Underestimated Risk Factor

**DOI:** 10.3390/cimb45020083

**Published:** 2023-02-03

**Authors:** Péter Mauchart, Réka Anna Vass, Bernadett Nagy, Endre Sulyok, József Bódis, Kálmán Kovács

**Affiliations:** 1 National Laboratory on Human Reproduction, University of Pécs, 7624 Pécs, Hungary; 2 Department of Obstetrics and Gynecology, Medical School, University of Pécs, 7624 Pécs, Hungary; 3 MTA-PTE Human Reproduction Scientific Research Group, 7624 Pécs, Hungary; 4 Faculty of Health Sciences, Doctoral School of Health Sciences, University of Pécs, 7621 Pécs, Hungary

**Keywords:** IVF, antioxidants, light protection, embryo, oocyte, sperm

## Abstract

Based on current findings, the presence of oxidative stress has a significant impact on the quality of gametes and embryos when performing assisted reproductive techniques (ART). Unfortunately, in vitro manipulation of these cells exposes them to a higher level of reactive oxygen species (ROS). The primary goal of this review is to provide a comprehensive overview of the development of oxidative stress in female and male reproductive systems, as well as in the case of the pre-implantation embryo and its environment. This review also focuses on the origins of ROS and the mechanisms of oxidative stress-induced damage during ART procedures. A well-known but underestimated hazard, light exposure-related photo-oxidation, is particularly concerning. The effect of oxidative stress on ART outcomes, as well as the various strategies for preventing it, are also discussed. We emphasize the role and significance of antioxidants and light protection including forms, functions, and mechanisms in the development of gametes and embryos in vivo and in vitro.

## 1. Introduction

In the past few decades, the study of the role of oxidative stress (OS) in reproductive health has become more and more popular. Oxygen is a key element of aerobic life, and oxidative metabolism represents an essential supply of energy. All multicellular aerobic organisms require molecular oxygen for their survival. The electron configuration of oxygen is special, as it has two unpaired electrons in different orbits in its outer shell, which makes it prone to forming radicals. The reduction of molecular oxygen (O_2_) yields superoxide (^•^O_2_^−^), which is the precursor of most other reactive oxygen species (ROS) [1,2]. ROS originate from the mitochondria along with other superoxides, and have a complex role in numerous cell signaling pathways that control cell proliferation rates and other cellular activities, such as molecular responses to hypoxia [3,4,5].

Moreover, ROS have a significant effect on the oxidative modification of many macromolecules such as proteins, receptors, ion channels, or transcription factors [6,7]. Consequently, a small amount of ROS are essential for the natural cell functions [8]. There are two types of oxidants that can produce free radicals: endogenous and exogenous oxidants. ROS are highly reactive and thus unstable, but they can be stabilized by acquiring electrons from nearby molecules (e.g., lipids, proteins, nucleic acids), resulting in cell damage and pathology [9,10,11]. Therefore, OS can cause lipid peroxidation and DNA and protein damage. In a healthy environment, every aerobic cell has a defense system against ROS, there is a precisely adjusted balance (homeostasis) between prooxidants and antioxidants (AOX). Superoxide anions (O_2_^−^), hydroxyl radicals (OH^−^), peroxyls (ROO), alkoxyls (RO), and hydroperoxyls (HO_2_) have the biggest biological importance among ROS. Enzymatic and non-enzymatic antioxidants are the two types of antioxidants that can be found in the body under normal conditions. The most prominent enzymatic antioxidants are catalase (CAT), glutathione peroxidase (GSH-Px), glutathione reductase (GSH-R), and superoxide dismutase (SOD), which can cause the reduction of hydrogen-peroxide (H_2_O_2_) to alcohol and water. For example, the non-enzymatic antioxidants are vitamins such as vitamin A, C, E, plant polyphenols, carotenoids, and glutathione or zinc [12]. These antioxidants are also treated as dietary supplements and synthetic antioxidants. By attaching to these harmful molecules, antioxidants reduce the effects of oxidants. Antioxidants, on the other hand, are beneficial at low concentrations and can function as oxidants at higher amounts. [13]. Therefore, the role of OS in male and female fertility is of particular importance. Therefore, in this report it will be briefly outlined.

## 2. Effect of Oxidative Stress on the Reproductive Tract of Males

### 2.1. Sources of ROS in Sperm

Spermatozoa obtain energy from two major metabolic pathways: glycolysis, which occurs in the main part of the flagellum, and oxidative phosphorylation, which occurs in mitochondria located in the flagellum’s midpiece [14]. There is no evidence that the process of the tricarboxylic acid cycle plays a role in adenosine triphosphate (ATP) production. By obtaining approximately 30 molecules of ATP by oxidizing one molecule of glucose, oxidative phosphorylation is the more efficient pathway. During glycolysis only two molecules of ATP are gained from each molecule of glucose.

### 2.2. Physiological Role of ROS in Sperm

**Figure 1:**
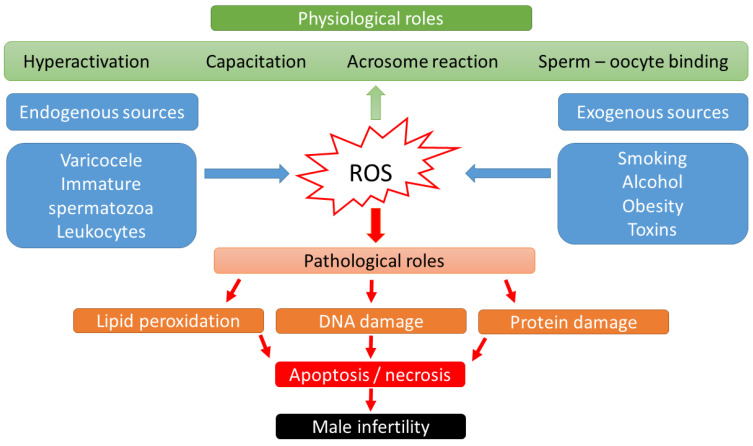
Scheme of physiological and pathological effects of reactive oxygen species (ROS) on male fertility.

Over the last two decades, it has become known that ROS may have a dual role in sperm function [15]: low ROS levels promote numerous intracellular processes leading to oocyte fertilization, whereas higher ROS levels may lead to DNA damage and embryo loss [16,17] (Figure 1). Physiological levels of ROS have an impact on diverse signaling pathways that regulate physiological redox-sensitive activities, since ROS generally mediates cell proliferation, apoptotic pathways that regulate the cell cycle and programmed cell death [18]. In order to fertilize the oocyte, spermatozoa must undergo various processes in the epididymis, such as sperm maturation, and in the female reproductive tract after ejaculation, such as hyperactivation, capacitation, and the acrosome reaction. During sperm maturation, ROS levels in seminal fluid have been shown to be critical for membrane protein rearrangements, enzymatic modulations, and nuclear remodeling [19]. In the case of nuclear remodeling, in addition to the inevitable replacement of histone proteins with smaller protamines [20], ROS also play a non-negligible role in stabilizing disulfide bonds to maintain chromatin stability [19]. During the ROS-mediated process of hyperactivation, the motility pattern of sperm changes significantly [21]. The hyperactivated sperm is characterized by a high-amplitude, asymmetric beating pattern of the sperm tail (flagellum). The biochemical background was recently described by Dutta et al., 2020 [22]: Calcium ions (Ca^2+^) and ROS (superoxide, O_2_^−^) mediate activation of adenylate cyclase (AC) and increased production of intracellular cyclic adenosine monophosphate (cAMP), which activates protein kinase A (PKA) [23]. Increased levels of PKA lead to activation of protein tyrosine kinase (PTK), resulting in phosphorylation of serine (Ser) and tyrosine (Tyr) residues. These steps lead to the essential changes in the cytoskeleton of the flagellum and the fibrous sheath of the axoneme. In parallel with hyperactivation, the increased level of phosphorylated tyrosine residues (P-Tyr) also leads to the process of capacitation, when the sperm cell prepares for the acrosome reaction. The biochemical features of the acrosome reaction overlap with those of capacitation. Both processes involve the influx of Ca^2+^ and increased levels of cAMP, PKA, and PKC. However, molecules such as phospholipase A2 (PLA2) are also involved in the acrosome reaction. PLA2 is activated in spermatozoa by progesterone secreted from the cumulus cell and cleaves intact phosphoglycerolipids into free fatty acids and lysophospholipids, increasing the fluidity of the sperm plasma membrane in preparation for sperm–oocyte fusion [24]. Then, the capacitated sperm binds to a glycoprotein of the zona pellucida, the process of which leads to oocyte penetration, and sperm head decondensation [24,25]. ROS have a low-level role as a second messenger in these fertilization processes.

### 2.3. Pathological Role of ROS in Sperm

High levels of ROS biological markers were identified in semen samples from 25–40% of infertile men [26]. Thus, male sub- and infertility are frequently related to OS. The source of the OS could be categorized into endogenous and exogenous factors. Lifestyle habits, such as alcohol intake, smoking, contact with toxic materials (radiation or environmental pollutants), or pathological abnormalities such as obesity, varicocele, stress, and aging have been connected with elevated production of adipokines, cytokines, and high levels of ROS in seminal plasma [27]. Supraphysiologic ROS levels have been correlated with the presence of leukocytes in seminal fluid, as well as a high percentage of morphologically abnormal spermatozoa [28] or immature spermatozoa with cytoplasmatic droplets containing a high number of enzymes [24,29]. In cases of leukocytospermia (no. of leukocytes ≥ 1 × 10^6^/mL), an increase in extracellular ROS generation is particularly evident, as the antioxidant protection of the seminal plasma becomes insufficient. Activated leukocytes can produce 100 times more ROS than non-activated leukocytes during inflammation or infection [30].

In the context of ART, gametes are exposed to in vitro modification, which regularly exposes these cells to OS [31]. However, leukocytes can be removed from sperm suspensions using procedures such as density gradient centrifugation (DGC) or swim-up; however, using these techniques without serum albumin has been related to sperm damage in several studies. In the absence of albumin, free radicals created by mitochondria during centrifugation trigger membrane lipid peroxidation and DNA damage [32,33]. This damage could be caused by peroxide produced by manganese superoxide dismutase (MnSOD) from superoxide radicals in tightly packed sperm pellets. It is released from damaged mitochondria and causes lipid peroxidation of the cell membrane, depolarization of mitochondria, decreased ATP synthesis, and sperm motility [34,35]. Advanced selection approaches such as microelectrophoresis, Zeta potential, and microfluidic technologies could be used to reduce the induction of OS and the resulting increase in DNA damage. However, such technologies are still used infrequently in clinics [36].

### 2.4. Effects of OS on Sperm Functions

Lipid peroxidation of the sperm membrane is the major mechanism of ROS-induced sperm destruction, which leads to infertility. Because their cell membrane and cytoplasm contain large quantities of polyunsaturated fatty acids, spermatozoa are sensitive to ROS [37]. This lipid peroxidation reduces sperm motility, likely due to a rapid loss of intracellular ATP, leading to a reduction in axonemal protein phosphorylation, which might result in decrease in motility and, subsequently, sperm immobility [38].

Furthermore, ROS may decrease sperm viability and enhance morphological defects in the mid-piece [39]. In case of DNA damage, the production of basis-free sites, deletions, frameshifts, DNA cross-links, chromosomal rearrangements, and DNA strand breaks could occur [40,41,42]. One study revealed that a 25% increase in ROS level in seminal plasma led to a 10% increase in DNA fragmentation [43]. These changes can cause the start or stop of gene transcription, accelerated degradation of telomeric DNA, epigenetic changes, replication mistakes, and GC-to-TA transversions [44]. In the case of the spermatozoa, only one base excision repair (BER) enzyme has been described, which is the 8-oxoguanine DNA glycosylase 1 (OGG1). Therefore, the DNA repair potential of spermatozoa is strongly limited, and much more exposed to ROS than other gametes [45]. However, cells normally rely on a variety of intrinsic and extrinsic antioxidant systems to neutralize high amounts of ROS. Enzymatic antioxidants such as SOD, catalase, and thiol peroxidases, as well as nonenzymatic antioxidants such as glutathione, are forms of endogenous antioxidants. Extrinsic antioxidants, on the other hand, are micronutrients such as vitamin C, vitamin E, L-carnitine, N-acetyl cysteine, and trace elements such as selenium or zinc [2] that must be provided from external sources in order to maintain a balance between oxidation and reduction (antioxidation) in any living cell of the body [1].

### 2.5. Methods Used to Counteract OS Effects

Although there are some contradictory reports that oral consumption of antioxidant-rich medication seems to improve sperm functional parameters such as motility and concentration, as well as decrease DNA damage, there is insufficient evidence that antioxidant consumption has a significant effect on the improving of fertility rates and live birth rates [46]. Furthermore, it is dependent on the type of antioxidants, the duration of treatment, and even the diagnosis of the man’s fertility, among further aspects [46]. A recent study discovered a significant effect of three-month lifestyle changes combined with oral antioxidant intake on DNA fragmentation index (DFI), but no effect on sperm concentration or total motile sperm count [47]. ‘Reductive stress’ refers to a change in the redox levels of the body to a more reduced state. According to reports, reductive stress is just as harmful as oxidative stress [48].

## 3. Effect of Oxidative Stress on the Reproductive Tract of Females

### 3.1. Physiological Roles of OS in Females Reproductive Tract

OS is the result of enormous ROS contributing to oocyte aging and several disorders affecting female reproduction. OS is considered to have cytotoxic effects by initiating the peroxidation of membrane phospholipids and altering nucleic acids, lipids, and proteins. These processes result in changes in the cellular physiology, including apoptosis, increased membrane permeability, even the total loss of membrane integrity, decreased enzyme activity, structural DNA damage, mitochondrial alterations, and ATP depletion [38,49,50]. The evolved free radicals may alter the oocyte, sperm, and embryos in their follicular and tubal fluids and peritoneal fluid microenvironments, and through these changes influence reproductive outcomes [51,52]. The imbalance of the redox system affects the female reproductive organs and results in oxidative stress, which impacts the function of the ovaries, the salpinx, the placenta, and the uterus. ROS may act as important mediators in hormone signaling, oocyte maturation, ovarian steroidogenesis, ovulation, luteolysis, luteal maintenance in pregnancy, implantation, compaction, blastocyst development, germ cell function, and corpus luteum formation [38].

### 3.2. Pathological Roles of OS in Female Reproductive Tract

It is also known that OS causes lipid damage and inhibits protein synthesis and TP depletion. These processes were described in the background of common obstetrical situations, e.g., the preterm premature rupture of the membranes, since oxidant stress caused by elevated ROS levels and simultaneous antioxidant depletion may damage collagen, resulting in premature membrane rupture [53]. Hypoxia causes altered placental function, leading to preeclampsia and fetal growth restriction. Particularly, in late gestation, elevated oxidative stress was detected in pregnancies complicated by diabetes, intrauterine growth restriction, and preeclampsia in association with increased trophoblast apoptosis and deportation and impaired placental vascular reactivity. OS was detected by increased lipid peroxides and isoprostanes and declined antioxidant expression and activity [54].

Normal endometrium has decreased SOD activity and increased ROS levels in the late secretory phase. An OS-induced autoantibody titer increase was detected in the peritoneal fluid of patients diagnosed with endometriosis. Elevated lysophosphatidyl choline—a known chemotactic factor of T lymphocytes—was observed in the same peritoneal fluid samples [55]. Expression of SOD, Mn and Cu-Zn dismutases, lipid peroxides, or glutathione peroxidase were detected in normal ovarian cycling [9,56]. Ovarian steroidogenesis is presumably associated with OS, while their expression is correlated with Ad4-binding protein—which serves as a general regulator of steroidogenic P450 genes—and superoxide dismutase expression [56]. In cell cultures, hydrogen peroxide resulted in reduced progesterone and estradiol hormones [57]. Suzuki et al. [56] also hypothesize that luteal Cu-Zn SOD has a supportive function in pregnancy.

OS influences the placenta, as Watson and coworkers [58] found syncytiotrophoblast damage in elevated oxygen levels resulted in microvilli decrease on their surface and a decrease in mitochondria. In another work, the same investigators proved that syncytiotrophoblast cells express antioxidants in early pregnancy [58,59].

Oxidative stress is an undeniable factor in the pathophysiology of other obstetrical diseases including polycystic ovarian disease, different fetal embryopathies, or intrauterine growth retardation, which have been associated with increasing OS. One factor in the background of these disorders is the activation of redox-sensitive transcription factors, such as p53 or NF-κB through the activation of different proinflammatory cytokines, such as interleukin-6 (IL-6), IL-18, or tumor necrosis factor (TNF-alfa), described in polycystic ovary syndrome (PCOS) [60]. Another process is protein oxidation, e.g., PCOS patients had higher plasma-advanced oxidation protein products in serum samples compared to control women [61]. The opening of ion channels has been described in the background since the increased ROS presence leads to Ca^2+^ ion release from the endoplasmic reticulum and other stores and this dysregulation leads to follicular arrest and reproductive or menstrual dysfunction [62]. Systemic endovascular inflammation detected in preeclampsia is caused by the dysfunction of maternal endothelial cells, leading to proteinuria and hypertension [63]. Oxidative stress participates in the development of intrauterine growth restriction (IUGR) through elevated levels of malondialdehyde, xanthine oxidase in maternal plasma, umbilical cord plasma, and placental tissue compared to the control group. An increased superoxide dismutase activity in maternal plasma and cord blood samples and an elevated glutathione peroxidase activity in maternal plasma and placental tissue were measured, while catalase activity was decreased in cord blood and placental tissue samples in IUGR groups [64].

An increased level of OS has been reported in women of advanced age undergoing in vitro fertilization (IVF) treatment [65]. An increased presence of OS in follicular fluid leads to ovarian senescence. Oocyte maturation is an essential process during IVF and intracytoplasmic sperm injection (ICSI). OS is altered in various reproductive processes such as oocyte maturation and folliculogenesis and is detrimental to natural and assisted fertility. Ovulation is essential for reproduction and is initiated by the luteinizing hormone surge; however, the overabundance of inflammatory precursors following LH surge generates ROS [66]. A previous study showed higher total antioxidant capacity in infertile women aged 30–39 years compared to pregnant women of the same age [67]. The erroneous oocyte mitochondria are responsible for the increased in vivo ROS levels. Age-related processes seem to be associated with ROS aggregation and mitochondrial dysfunction [68]. The repeated ovulation may accumulate, resulting in inflammatory changes in the ovary and promoting oxidative damage [66,69]. Presumably, the elevated presence of ROS disrupts the prooxidant–antioxidant balance in peritoneal fluid and leads to infertility in women, causing damaged or degenerated cytoskeleton fibers. Higher ROS levels directly affect the ovum after its release from the ovary, the development of zygote/embryo, or damage the spermatozoa [70]. Previous investigations comparing the presence of ROS in peritoneal fluid samples of women undergoing laparoscopy under infertility assessment and fertile women operated with tubal ligation showed elevated ROS levels in infertile patients [50]. In the same study, investigators found that infertile patients had significantly reduced levels of antioxidants such as vitamin E and glutathione. Reduced ability to eliminate ROS to neutralize toxic effects causes uncompensated balance [50] and the proposal to use antioxidant treatment in clinical practice. Free radical-induced damage may be partly involved in the age-related fall of follicle reserves [71]. IVF patients in advanced reproductive age may show reduced expression of genes responsible for the dissolution of ROS [65], such as a decline in the SOD1 or SOD2, catalyzing mRNA composition, confirming that reproductive aging may downregulate the protective gene expression of granulosa cells [72]. High follicular fluid ROS levels are associated with negative IVF outcomes, particularly in smokers [38]. There is a growing interest in the examination of OS in the female reproductive system, since it may be a crucial point in investigating the reason for infertility.

Not only are the maternal functions affected by OS and AOX systems. The sensitive fetus is constantly responsive to the maternal milieu, and previous works prove that OS leads to several pregnancy-associated disorders affecting fetal intrauterine development. Moreover, the placenta does not prevent the infiltration of harmful factors and substances from the maternal circulation to the fetus. Previous investigations have also described that environmental toxins affecting the mother can be shifted directly to the fetus during pregnancy [73], leading to the activation, and, in this way, the potential programming of the AOX defense system. It is necessary to resolve whether the epigenetic modification of the AOX system is possible. It is known that a high-fat diet induces epigenetic changes in the fetal epigenome and alters AOX genes, such as hepatic Pon1 gene, a known antioxidant. It is possible that the AOX system can be epigenetically programmed in utero, since investigators observed that the liver of fetuses whose mothers followed an HF diet during their pregnancy did not later develop obesity [74]. Although the supplement and vitamin market has developed exponentially worldwide [75], clinical trials did not clearly prove their beneficial role during fertility treatments. A previous review summarized and analyzed the results of 63 trials focusing on the effects of different antioxidants (L-arginine, vitamin E, myo-inositol, D-chiro-inositol, carnitine, selenium, vitamin B complex, vitamin C, vitamin D + calcium, CoQ10, and omega-3 polyunsaturated fatty acids) with the participation of 7760 women. The authors concluded that trials provide limited evidence about the beneficial and protective effects of antioxidant use [76]. An association was found between antioxidant use and in the development of clinical pregnancy rates among women with PCOS [77]. Vitamin D supplementation was beneficial in menstrual dysfunction [78]. Application of micronutrients positively influenced the pregnancy rate and live birth in case of IVF pregnancies [76,79].

## 4. Oxidative Stress in Pre-Embryos and Their Surroundings

Energy production, including ATP molecules, starts in parallel with embryonic development. During normal aerobic metabolism-related embryonic development, three free radicals are known to be present: hydrogen peroxide (H_2_O_2_), superoxide anion (O_2_^−^), and hydroxyl radical (OH^−^) [80]. The effect of free radicals on embryonic development could be considered complex, as these molecules have a diverse impact, such as deterioration of cell promotion, depending on the number of free radicals, starting from the stage of development (fertilization, cleavage state, compaction, blastulation) and the environment (in vivo or in vitro). ROS are produced by spermatozoa and leukocytes during fertilization, as well as during processes such as sperm-induced oocyte activation and the activation of the embryonic genome [81]. OS can also arise all through in vitro embryo production (IVP), starting from in vitro maturation to the progress of embryo development, as the protective antioxidant mechanisms that function in vivo are absent in vitro [82]. As Argawal et al. [31] reviewed, the metabolic processes of the embryos are causing an increase in the amount of highly toxic ammonia, which may cause damages in the cells through ROS overproduction.

### 4.1. Sources of ROS during ART

In conventional IVF, the potential cellular origin of ROS differs from those of cells fertilized by ICSI [83]. The 4–5 oocytes in each dish, the few thousand cumulus cells, and the approximately 150–200 × 10^6^ spermatozoa used for insemination in conventional IVF can all produce ROS during co-incubation. Diseases such as PCOS [84] or endometriosis [85] are characterized by elevated levels of ROS in the oocyte environment. In these cases, it is suggested to perform ICSI because cumulus cells are no longer a possible source of ROS since the incubation starts after the oocytes have been depleted of all cumulus cells. Spermatozoa and their injection into the oocytes are two potential biological sources of ROS in the ICSI setup. However, if ICSI is performed in a male indication with a healthy female partner, it is conceivable that the absence of cumulus cells may have the opposite effect, i.e., a reduction in the oocyte’s resistance to ROS [86]. In most IVF labs, rather extended insemination periods (14–16 h) are the standard practice. Although prolonged exposure time of oocytes to spermatozoa might cause oxidative damage [87], several groups have looked into shortening the exposure time. The outcomes have been inconsistent. Several researchers stated that short co-incubation of gametes in IVF (usually 2–4 h) had positive results [88,89], whereas others claimed the contrary [90].

**Figure 2:**
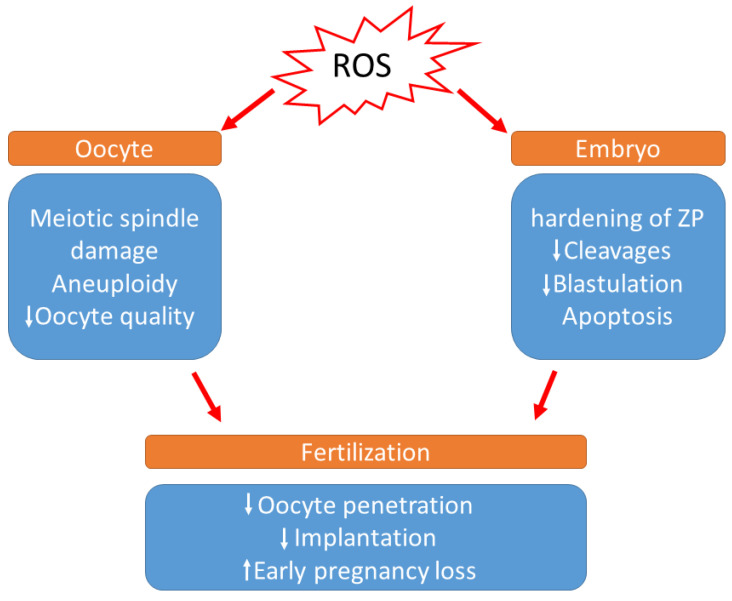
Effects of increased levels of reactive oxygen species during IVF. (ROS: Reactive oxygen species, ZP: zona pellucida).

During in vitro development, the absence of non-enzymatic antioxidants in the environment surrounding the oocytes, the difference in O_2_ concentration between in vivo and in vitro conditions, visible light, and culture media additives can also contribute to ROS generation. According to a previous study, preimplantation embryos are especially vulnerable to conditions that trigger OS [91]. It has been demonstrated that a direct association occurs between increased ROS concentration and programmed cell death (apoptosis), resulting in the degree of embryo fragmentation or the poor rate of blastocyst development [92,93] (Figure 2). Cell necrosis causes swelling and rupture of the cell membrane, whereas fragmentation causes the cell to condense and divide into numerous fragments, resulting in cytoplasmic condensation and condensed nuclei, which are referred to as apoptotic bodies [93]. Moreover, increased ROS levels in the embryo cause mitochondrial changes, cell blockage, ATP depletion, and apoptosis. Mitochondrial DNA is more vulnerable to mutation due to a lack of histones, which also serve to reduce ROS. Defective mitochondrial DNA in embryos can cause metabolic malfunction and, as a result, disrupt in embryo development. These changes may have a variety of effects, including embryo development retardation and arrest, metabolic dysfunction, and possibly apoptosis [71]. According to Várnagy et al. [94], the level of 8-hydroxy-2′-deoxyguanosine (8-OHdG) in the follicular fluid, a biomarker of oxidative DNA damage, has a detrimental impact on the number of good quality embryos. DNA damage caused by oxidative stress may potentially result in early pregnancy loss [95]. Exposure to ROS results in the hardening of the zona pellucida and can weaken the implantation ability of embryos [96].

The importance of ROS is decreased at the blastocyst stage because the embryo shifts from oxidative phosphorylation to aerobic glycolysis for sustenance protein synthesis and ion transport systems [97,98]. A low level of ROS produced by embryos, on the other hand, is required for development regulation [99].

The reproductive tract not only produces oocytes, but also protects gametes and the embryo from visible light exposure. During the processes of assisted reproduction, (retrieval of oocytes, preparation of the sperm, IVF or ICSI procedure, incubation and microscopic examination of formed embryos, embryo transfer), gametes, zygotes, and embryos are subjected to a variable spectrum of light from different sources, including safety cabinets, microscopes, or time-lapse imaging cameras [100,101].

### 4.2. The Effect of Light Exposure of Gametes and Embryos

Subdued and filtered light using red filters on laboratory lamps and UV or infrared filters in microscopes to eliminate white and UV light exposure, throughout all work stages, to sperm cells, oocytes, and embryos, resulted in better embryo quality [102]. Because light generates reactive oxygen species (ROS), oxidative stress is considered one of the plausible causes at the origin of the embryonic lesion. The harmful effects of light are associated with the generation of H_2_O_2_ in peroxisomes and mitochondria [103], activation of stress genes, or direct DNA damage via ionization [100].

The toxic effects of UV light have long been known for cells. Previous studies showed that not only UV radiation, but also visible light is toxic to mammalian cells [104,105]. However, the toxic effects of visible light (400–800 nm) are less commonly recognized. Still, several studies confirm the harmful effects of visible light on oocytes, sperm, and embryos [101,104,106].

Furthermore, the detrimental effects of visible light depend on the wavelength. Based on studies of light radiation, stress gene activation and DNA damage in embryos can also be triggered. Blue light (400–500 nm) is declared to be orders of magnitude more deleterious than longer wavelengths of the visible spectrum [101,104].

Light-generated ROS formation takes place in cellular flavins that absorb light and in membranal chromophores. ROS generation can cause mitochondrial dysfunction and cellular damage [101] and changes the membrane redox state, which might lead to membrane channel opening [105].

Based on the above, using light filters may reduce detrimental environmental factors in an IVF laboratory. Bognar et al. [105] showed that white light exposure reduced the implantation potential of in vitro cultured mouse embryos. However, if a red optical filter was used the harmful effect of light was reduced. Our recent human study shows how essential it is to reduce the detrimental effects of illumination, thereby preserving the number of viable embryos and minimizing embryo loss during IVF and ICSI [102].

### 4.3. OS in the Embryo Culture Medium

**Figure 3:**
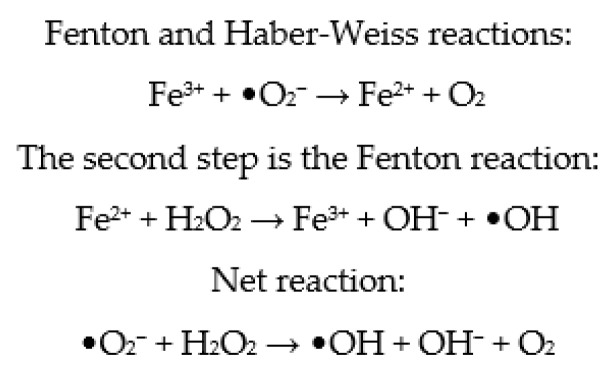
Fenton and Haber–Weiss reactions.

Metallic ions in culture media, such as Fe^2+^ and Cu^2+^, have the ability to speed up cell ROS production by participating in Fenton and Haber–Weiss processes (Figure 3) [80]. As a result, depending on the composition of commercial embryo culture media, variable levels of ROS are generated. However, while endogenous ROS are formed by embryo metabolism, exogenous ROS are formed spontaneously by buffers and different types of additives in the culture medium. Consequently, additional, exogenous antioxidants appear to be required [107]. Therefore, antioxidants are generally added to the embryo culture medium, ensuring that the oxidant and antioxidant balance in the embryos is maintained [82]. However, it has been established that the formation of ROS is higher in more complex culture media compared to simple media [108,109]. To enhance embryo quality and viability in vitro, it appears that the composition of the embryo culture medium must be optimized. It has been suggested that antioxidants be added to the maturation medium to reduce the danger of oxidative stress and subsequent DNA damage [82].

ROS inducers can also be found among media additives. Because of its significant antioxidant capabilities, serum albumin is an important addition [110]. Serum preparations, which are often added to culture media, include high quantities of amine oxidase, which results in a rise in H_2_O_2_ production [111].

### 4.4. Methods Used to Counteract OS Effects

Human serum albumin (HSA) is currently being used to improve human embryo culture media as a protein supplement with antioxidant properties [112,113]. Copper ions attach to particular binding sites in albumin and have the ability to speed up the decomposition of free radical processes. Pool and Martin [114] were the first to use HSA in human embryo culture conditions, demonstrating that albumin increased embryo growth. For fertilization and embryo development, rHSA was found to be as effective as HSA. Furthermore, employing rHSA in IVF may reduce contamination and the transfer of plasma-derived contaminants. Due to the expensive cost of manufacture, rHSA is not used extensively as an additive in human embryo culture media [2]. Since the primarily discovered add-on with antioxidant effect, it has been found that many more molecules are proved to be effective in reducing OS in embryonic culture droplets. Aitken reviewed in 2020 [115] that molecules such as alpha-lipoic acid, hypotaurine and N-acetyl cysteine, 9-cis-retinoic acid, coenzyme Q10, melatonin, rosmarinic acid, and citrus flavonoids or hesperetin are now commonly used by the manufactures of embryo culture media.

A study on antioxidants is required to determine optimal supplementation levels for human embryo culture media, because many antioxidants have positive effects on embryo growth during ART. Antioxidants are an effective therapeutic method. It is, nevertheless, difficult to identify a superior or optimal culture medium from the others [116], even if we consider that the ROS levels of various commercial cultural media, even those produced by the same company, could differ significantly [116]. As Yang et al. [93] reviewed as a result, adding free radical scavengers and metal chelators, such as SOD, transferrin, ethylenediaminetetraacetic acid (EDTA), and thioredoxin, to the culture media at a low oxygen concentration may lead to improved embryo development.

## 5. Conclusions

In conclusion, mild levels of ROS have a non-negligible effect on the physiological maturation processes of gametes. As signaling molecules, they have importance in the regulation of cell proliferation rates and apoptosis, as well as in the modulation of gene expression pathways. ROS play a crucial role in the normal functioning of spermatozoa, oocytes, and in the development of the preimplantation embryo. In contrast, OS, due to elevated ROS levels, is one of the most important disorders that can lead to sub- and infertility in both men and women. Whereas in a healthy environment, there is a precisely adjusted balance between ROS and antioxidants. The elevated levels of ROS are extremely harmful due to the damage of lipids, DNA, or proteins. Among the potential ROS sources during ART, we highlighted the importance of reducing photo-oxidative stress by using light filters that can reduce harmful environmental factors in an IVF laboratory. Not only the recognition of the molecular networks between pro- and antioxidant pathways, but also the determination of the biological conditions that either predispose to the bioaccumulation of ROS or promote the biodestruction of oxygen-derived free radicals, could be helpful in undertaking various efforts related to the practical application of assisted reproductive technologies (ARTs) in different mammalian species. These efforts include the improvement of both developmental competence and quality-related parameters of in vitro-produced embryos generated by gamete coincubation or intracytoplasmic sperm injection (ICSI)-mediated IVF [117,118,119,120] or by somatic cell nuclear transfer (SCNT)-based cloning [121,122,123].

## Data Availability

Not applicable.
